# Systemic Inflammation and Its Interaction with Alzheimer’s Disease Pathology in Accelerating Cognitive Decline

**DOI:** 10.1093/geroni/igaf122.4294

**Published:** 2025-12-31

**Authors:** Diefei Chen, Keenan Walker, Marilyn Albert, Jeremy Walston, Jacqueline Langdon, Alden Gross

**Affiliations:** Johns Hopkins Bloomberg School of Public Health, Baltimore, Maryland, United States; National Institute on Aging, Baltimore, Maryland, United States; Johns Hopkins School of Medicine, Baltimore, Maryland, United States; Johns Hopkins School of Medicine, Baltimore, Maryland, United States; Johns Hopkins School of Medicine, Baltimore, Maryland, United States; Johns Hopkins Bloomberg School of Public Health, Baltimore, Maryland, United States

## Abstract

Systemic inflammation may accelerate cognitive decline related to Alzheimer’s disease (AD), yet underlying mechanisms remain unclear. We tested whether systemic inflammatory markers are associated with cognitive decline and whether they interact with AD pathology (amyloid, tau) and neuroinflammation (GFAP) to accelerate cognitive decline. We analyzed 161 participants from the BIOCARD study, who were cognitively unimpaired at baseline, with the first biofluid collection at mean age of 59.3 years (SD = 6.2), median of 3 samples (range 2–5) over 19.5 years on average. Blood-based systemic inflammatory markers were: IL1β, IL6, IL8, TNFα, TNFR1, CRP. Baseline Aβ42/40, ptau181, and GFAP were assayed in both CSF and blood. Linear mixedeffects models related (1) baseline systemic inflammatory levels, (2) withinperson change in each systemic inflammatory marker, and (3) interactions between baseline systemic inflammation and Aβ, ptau181, or GFAP to change in a general cognitive factor score, adjusting for age, sex, education, and vascular risk. Systemic inflammatory markers alone were not associated with faster cognitive decline. However, significant interactions indicated steeper cognitive decline when systemic inflammation cooccurred with AD pathology or neuroinflammation. Using CSF markers, faster decline was seen for CRP×Aβ42/40, TNFR1×ptau181, and IL1β×GFAP. Using blood-based markers, similar patterns emerged for CRP×Aβ42/40; IL8, TNFα, TNFR1×ptau181; IL8, TNFR1×GFAP. Systemic inflammation did not predict cognitive decline on its own but amplified the adverse effects of amyloid, tau, and GFAP. These findings support a synergistic model in which systemic inflammation interacts with AD pathophysiology to accelerate cognitive decline and may aid in identifying individuals at elevated risk for targeted intervention.

